# Irreversible Effects of Affiliation With Delinquent Peers on Cyberbullying Perpetration Among Adolescents in Hong Kong: Moderating Effect of Student–Teacher Relationships

**DOI:** 10.1002/bsl.70030

**Published:** 2025-11-29

**Authors:** Yang Han, Ji‐Kang Chen

**Affiliations:** ^1^ Department of Social Work The Chinese University of Hong Kong Hong Kong China

**Keywords:** adolescents, affiliation with delinquent peers, asymmetric fixed‐effects regression, cyberbullying perpetration, irreversible effects, student–teacher relationships

## Abstract

Adolescent affiliation with delinquent peers can lead to offline delinquent behaviors; however, less is known about its effect on online delinquent behaviors, particularly cyberbullying perpetration. Furthermore, it is unclear whether the delinquent peer effect on cyberbullying perpetration can be reversed by reducing affiliation with delinquent peers. Using novel asymmetric fixed‐effects regression with two‐wave follow‐up data from Hong Kong (*N* = 356) to control for observed time‐variant and observed and unobserved time‐invariant confounders, we found that increased affiliation with delinquent peers was associated with increased cyberbullying perpetration, whereas decreased affiliation with delinquent peers was not associated with cyberbullying perpetration. Additionally, improving student–teacher relationships mitigated the effect of increased affiliation with delinquent peers on cyberbullying perpetration. Therefore, the effect of affiliation with delinquent peers on cyberbullying perpetration may be irreversible. Nevertheless, interventions and policies can aim to enhance student–teacher relationships to alleviate the delinquent peer effect on cyberbullying perpetration.

## 1 Introduction

Adolescent affiliation with delinquent peers has long been considered a risk factor for offline delinquent behaviors (Akers et al. 1979; Chen, Yang, Lin, et al. 2023; Haynie 2001; Rodriguez et al. 2019; Thornberry et al. 1994). Additionally, a few recent studies have indicated that affiliation with delinquent peers is an important risk factor for online delinquent behaviors, particularly cyberbullying perpetration, the most common form of online delinquent behavior among adolescents (Kim et al. 2022; X. Wang et al. 2025). However, whether the effect of affiliation with delinquent peers on cyberbullying perpetration can be reversed remains unknown. That is, if increasing affiliation with delinquent peers leads to an increase in cyberbullying perpetration, will decreasing affiliation with delinquent peers necessarily decrease it? The above question is significant in that it concerns whether interventions aimed at reducing affiliation with delinquent peers are effective in reducing cyberbullying perpetration. However, previous studies cannot answer this question sufficiently, as they did not distinguish the effects of increased and decreased affiliation with delinquent peers on cyberbullying perpetration while controlling for unobserved time‐invariant confounders.

Specifically, previous studies (e.g., Kim et al. 2022; X. Wang et al. 2025) were based on an implicit *symmetric* effect assumption; that is, if cyberbullying perpetration increases X units with a one‐unit increase in affiliation with delinquent peers, then it will necessarily decrease the same X units when affiliation with delinquent peers decreases by one unit. For instance, if affiliation with delinquent peers is measured by how many kinds of delinquent behaviors one's peers have, and the regression coefficient of the association between affiliation with delinquent peers and cyberbullying perpetration is X with X being positive, the coefficient can be interpreted in two ways: if a student's peers have one more delinquent behavior, the frequency of the student's cyberbullying perpetration will increase by X. Alternatively, the coefficient also means that if a student's peers have one less delinquent behavior, the frequency of the student's cyberbullying perpetration will decrease by X. The above *symmetric* effect assumption implies that the effect of affiliation with delinquent peers on cyberbullying perpetration is reversible in that cyberbullying perpetration caused by affiliation with delinquent peers can be reduced by reducing affiliation with delinquent peers.

However, a decrease in affiliation with delinquent peers may not result in a corresponding decrease in cyberbullying perpetration, perhaps because the attitudes toward delinquency that adolescents learned from their delinquent peers still persist even if affiliation with delinquent peers decreases. In this sense, the effect of affiliation with delinquent peers on cyberbullying perpetration may be *asymmetric*, meaning that even if a one‐unit increase in affiliation with delinquent peers leads to an X‐unit increase in cyberbullying perpetration, a one‐unit decrease in affiliation with delinquent peers may *not* necessarily lead to the same X‐unit decrease in cyberbullying perpetration. If true, interventions aimed at reducing cyberbullying perpetration by decreasing affiliation with delinquent peers may not be as effective as expected.

To our knowledge, no previous study has separated affiliation with delinquent peers into its increased and decreased components and examined their separate associations with cyberbullying perpetration. In this sense, the results of previous studies could only be interpreted in the “symmetric” way. We employed a novel asymmetric fixed‐effects regression approach (Allison 2019) to separate affiliation with delinquent peers into its increased and decreased components and examine their separate associations with cyberbullying perpetration while controlling for the observed and unobserved time‐invariant confounders.

Furthermore, suppose that the effect of increased affiliation with delinquent peers on increased cyberbullying perpetration cannot be completely reversed by decreasing affiliation with delinquent peers. What factors can counteract the effect of increased affiliation with delinquent peers on cyberbullying perpetration? From a social relationship perspective, affiliation with delinquent peers is one part of an adolescent's social relationships. In the school setting, adolescents also form social relationships with teachers in addition to peers. Student–teacher relationships and peer relationships may not only independently affect adolescents' behaviors but also interact with each other in shaping their behaviors (Silver et al. 2005). Good student–teacher relationships could change students' attitudes toward bullying (C. Wang et al. 2015), which may counteract the effect of affiliation with delinquent peers on cyberbullying perpetration. However, empirical evidence in support of the above argument is lacking; hence, we intended to fill the research gap.

To summarize, we aimed to answer two questions in this study: (1) Is the effect of adolescent affiliation with delinquent peers on cyberbullying perpetration reversible? (2) If not, will improving student–teacher relationships mitigate the adverse effect of affiliation with delinquent peers on cyberbullying perpetration? We employed a novel asymmetric fixed‐effects regression approach (Allison 2019) to answer the above questions. The asymmetric fixed‐effects regression can divide an independent variable into its increased and decreased components while controlling for all observed and unobserved time‐invariant confounders (Allison 2019). By answering the above two questions using asymmetric fixed‐effects regression, we contribute to the literature in four ways. First, we bring the effects of affiliation with delinquent peers on offline delinquency into the online scenario. Second, we break the symmetric effect assumption on the effects of affiliation with delinquent peers on delinquency by examining the separated associations of increased and decreased affiliation with delinquent peers with cyberbullying perpetration. Third, we take the possible protective factor (namely, student–teacher relationships) into account to inform possible interventions and policies. Last, we provide more solid evidence than studies relying on between‐subject variations by employing the asymmetric fixed‐effects regression to eliminate potential confounding bias caused by unobserved time‐invariant factors.

### 1.1 Literature Review

#### 1.1.1 Influence of Affiliation With Delinquent Peers on Offline and Online Delinquency

For over one century, peer influence has been recognized as playing a pivotal role in shaping adolescents' delinquent behavior (McGloin and Thomas 2019). Several theories lay the foundations for the effects of affiliation with delinquent peers on adolescents' delinquency. Differential association theory (Sutherland 1947) posits that delinquent behaviors are learned through social relationships with peers that foster favorable attitudes toward law violation. Through this learning process, individuals not only learn how to conduct specific delinquent behaviors but also develop motivations and positive attitudes toward delinquent behaviors (Sutherland 1947). In this sense, adolescents who have more affiliation with delinquent peers may be more likely to form favorable attitudes toward delinquency and be more motivated to conduct delinquent behaviors, including cyberbullying perpetration. However, differential association theory does not explain when and how attitudes toward delinquent behaviors can be transformed into actual delinquent behaviors (Megens and Weerman 2012). Additionally, social psychology studies have long documented attitude‐behavior inconsistency (Glasman and Albarracín 2006; Wicker 1971; Yuan et al. 2023). Some criminology studies have also suggested that individuals' attitudes toward delinquent behaviors and their actions are weakly linked and not even consistent (Matsueda 1989; Megens and Weerman 2010).

Based on differential association theory (Sutherland 1947), social learning theory (Akers 1973; Akers et al. 1979) took a step further by incorporating operant (instrumental) conditioning. According to social learning theory (Akers 1973; Akers et al. 1979), peers also influence adolescents' delinquency through differential reinforcements in addition to differential association. If adolescents' peers show support in delinquent activities, the support works as reinforcement that increases their own delinquency. Furthermore, social learning theory also suggests that an individual may use the definitions favorable to delinquent behaviors to justify a committed offense (Akers 1998). Hence, peer influence could occur due to its influence on an individual's attitude as well as an individual's compliance resulting from group pressure (Megens and Weerman 2012). Warr and Stafford (1991) took a stronger position, suggesting that delinquent behaviors occur due to situational reasons, such as peer pressure, regardless of attitudes toward these delinquent behaviors.

Additionally, drawing on symbolic interactionism (Goffman 1959; Mead 1934), affiliation with delinquent peers could affect adolescents' expectations of others' reactions to delinquent behaviors through social interactions. Interactions with delinquent peers could lead to the expectation that peers will react favorably to delinquent behaviors. Therefore, once adolescents affiliate with delinquent peers, they tend to develop delinquent tendencies and engage in delinquent activities to meet the expectations of their delinquent peers.

A large body of research has provided evidence for the influence of delinquent peers in shaping offline delinquent behaviors, including substance use, alcohol drinking, and school bullying perpetration (Akers et al. 1979; Chen, Yang, Lin, et al. 2023; Giletta et al. 2021; Grant et al. 2019; Haynie 2001; Monahan et al. 2009; Rodriguez et al. 2019). Additionally, an increasing number of studies examining the effects of affiliation with delinquent peers on cybercrime and cyber deviance have emerged and suggest that affiliation with delinquent peers can lead to cyber delinquency (Holt et al. 2012; Marcum et al. 2014; Wissink et al. 2023; Yoo 2022), among which studies have demonstrated that affiliation with delinquent peers is a risk factor for cyberbullying perpetration (Kim et al. 2022; X. Wang et al. 2025).

Studies examining the effects of affiliation with delinquent peers on offline delinquent behaviors have employed diverse samples and various methods, enriching our understanding of the causal role of affiliation with delinquent peers in the offline setting (McGloin and Thomas 2019). In contrast, studies on the effects of affiliation with delinquent peers on online delinquent behaviors, particularly cyberbullying perpetration, are relatively scarce, hindering our knowledge of the applicability of the above theories in the online scenario. Additionally, the above scarcity limits our understanding of the causal role of affiliation with delinquent peers in online delinquency.

#### 1.1.2 Reversibility/Irreversibility of Influence of Delinquent Peers

According to the above theories and empirical studies, affiliation with delinquent peers leads to delinquent behaviors. Conversely, could the delinquent behaviors caused by affiliation with delinquent peers be reversed by reducing affiliation with delinquent peers? The foundational theories, such as differential association theory and social learning theory (Akers 1973; Akers et al. 1979; Mead 1934; Sutherland 1947), and their later extensions (Akers 1998; Bruinsma 1992) have not explicitly addressed how reducing affiliation with delinquent peers could reduce delinquent behaviors, including cyberbullying perpetration. Differential association theory also fails to consider personality traits (Djakovic and Rowlands 2024). Previous empirical studies have both supported (for a review, see Akers and Jennings 2019) and challenged or criticized the explanatory power of these foundational theories on criminal behavior and delinquency, as well as the imprecise definitions of concepts and related measurement difficulties (e.g., Brauer 2009; Brauer and Tittle 2012; Krohn 1999; Tittle et al. 1986). However, no previous empirical studies have distinguished between decreased and increased affiliation with delinquent peers and examined their separate effects on delinquent behaviors, including cyberbullying perpetration.

In the field of peer influence on delinquency, differential association and learning theories (Akers 1973; Akers et al. 1979; Sutherland 1947) emphasize the process of learning delinquency through delinquent peers. After learning from peers, individuals not only acquire the techniques to engage in delinquent activities but also develop attitudes and rationalizations that are favorable toward delinquency. More importantly, individuals can internalize the values regarding delinquency, and these internalized values can guide them to engage in delinquent activities even in the absence of peer pressure (Mulligan 1960). In this sense, as long as individuals have learned and internalized the values and attitudes favorable to delinquency, these values and attitudes can persist in leading to delinquency even if affiliation with delinquent peers decreases. Hence, reducing affiliation with delinquent peers may not necessarily reduce cyberbullying perpetration, resulting in the possible irreversibility of the effect of affiliation with delinquent peers on cyberbullying perpetration. However, research has predominantly overlooked the potential irreversibility mentioned above.

#### 1.1.3 Moderating Effect of Student–Teacher Relationships

Differential association theory (Sutherland 1947) emphasizes the existence of peer relationships. From a social relationship perspective, students also form social relationships with teachers in addition to peers in the school setting. Social relationships, including peer relationships and student–teacher relationships, can encourage individuals to conform to the norms and expectations of the relational groups (Haynie 2001). However, the norms and expectations embedded in the relationships with delinquent peers and with teachers may be different and even opposite; therefore, these two relationships may affect students' behaviors in different directions. Specifically, delinquent peers foster values and behaviors that are opposite to conventional social norms (Aseltine 1995). By contrast, teachers are role models of conventional social norms, and good relationships with teachers can lead students to conform to these conventional social norms (Obsuth et al. 2023). In this sense, good student–teacher relationships may counteract the effect of affiliation with delinquent peers on delinquency.

Additionally, differential association theory (Sutherland 1947) posits that affiliation with delinquent peers leads individuals to become delinquent by developing favorable attitudes toward delinquency (McGloin and Thomas 2019). In this sense, as good student–teacher relationships could make students' attitudes toward delinquency less favorable (C. Wang et al. 2015), these relationships may mitigate the effect of affiliation with delinquent peers on delinquency.

Numerous studies have examined the main effect of student–teacher relationships on delinquent behaviors (Chen et al. 2025; Gao et al. 2024; Obsuth et al. 2023; C. Wang et al. 2015). In contrast, few studies have examined the potential moderating effect of student–teacher relationships on the association between affiliation with delinquent peers and delinquent behaviors.

### 1.2 Present Study

**FIGURE 1:**
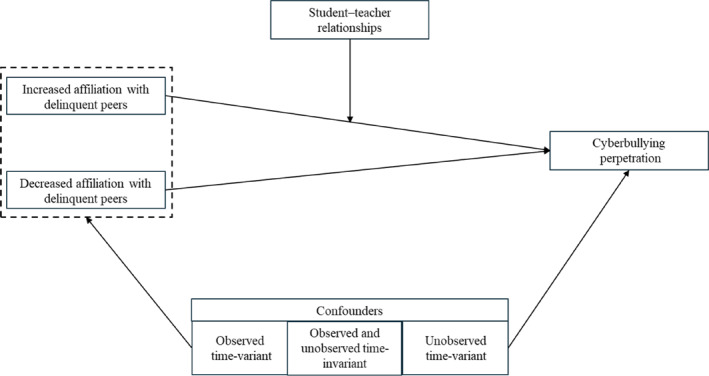
Analytical framework of the associations of increased and decreased affiliation with delinquent peers with cyberbullying perpetration and the moderating role of student–teacher relationships.

Based on the above literature review, we proposed our analytical framework in Figure 1. We hypothesized that after controlling for observed time‐variant factors and all observed and unobserved time‐invariant factors, (1) increased affiliation with delinquent peers would be associated with increased cyberbullying perpetration, which might not be reduced by decreased affiliation with delinquent peers, and (2) with the improvement of student–teacher relationships, the association between increased affiliation with delinquent peers and increased cyberbullying perpetration would became weaker.

## 2 Methods

### 2.1 Data and Participants

The data used in this study are from a two‐wave longitudinal survey conducted among junior high school students (Grades 7–9) and their homeroom teachers in Hong Kong. A cluster random sampling method was used to select student participants from all three administrative areas of Hong Kong, including Hong Kong Island, Kowloon, and the New Territories. Student participants answered questions about affiliation with delinquent peers, cyberbullying perpetration, and sociodemographic characteristics, among others. Additionally, the students' homeroom teachers answered questions regarding student–teacher relationships with each of the student participants. Student and teacher participants were surveyed twice, with a 9‐month interval in the same academic year. A total of 356 student participants, together with their homeroom teachers, completed the two‐wave longitudinal survey. The teacher data were matched with each student participant's data, and the 356 student participants comprised our sample.

### 2.2 Measurements

The dependent variable was cyberbullying perpetration. A total of six items from the traditional Chinese version of the Cyberbullying Scale (Chen and Chen 2020; Chen et al. 2023) were utilized to measure students' cyberbullying perpetration since the beginning of the semester. Students rated each of the items on a five‐point Likert scale from 1 (never) to 5 (seven or more times). The Cronbach's *α* coefficients for the six items were 0.60 and 0.88 in Waves 1 and 2, respectively. We calculated the total score of the six items, and a higher score indicated a higher level of cyberbullying perpetration.

The independent variable was affiliation with delinquent peers in school. Students were asked to indicate whether they had affiliated with friends in their school who exhibited any of the behaviors on a list of 11 delinquent behaviors (Chen, Yang, Lin, et al. 2023). Each of the 11 items was dichotomized with 1 = yes and 0 = no. The Cronbach's *α* coefficients for the 11 items were 0.77 and 0.84 in Waves 1 and 2, respectively. We calculated the total score of the 11 items, with a higher score indicating a higher level of affiliation with delinquent peers.

The moderator was student–teacher relationships. Student participants' homeroom teachers were invited to indicate their relationships with each of the student participants using the following four items from the Teacher–Student Relationship Inventory (Ang 2005): (1) “If this student needs help, he/she is likely to ask me for help.” (2) “The student turns to me for a listening ear or for sympathy.” (3) “I am happy with my relationship with this student.” (4) “I like this student.” Each of the four items was rated on a five‐point Likert scale from 1 (Strongly Disagree) to 5 (Strongly Agree). The Cronbach's *α* coefficients for the four items were 0.82 and 0.71 in Waves 1 and 2, respectively. We calculated the total score of the four items, and a higher score indicated a better student–teacher relationship.

The observed time‐invariant control variables were sex (male/female) and grade (7, 8, or 9), as each student's sex and grade remained constant from Wave 1 to Wave 2. The grade level did not change because, as mentioned above, the two waves of surveys were conducted in the same academic year. The time‐variant control variables were parental marital status and subjective family economic status, meaning that some students' parental marital status and subjective family economic status changed from Wave 1 to Wave 2. As suggested by family systems theories (Bowen et al. 2013; Bowen 1978), individuals are embedded in a family system and their behaviors are affected by family members. Additionally, family plays a central role in the primary socialization of children and adolescents, shaping their social behaviors (Whitbeck 1999). Therefore, we controlled parental marital status and subjective family economic status.

### 2.3 Statistical Analysis

We conducted descriptive statistics for the sample characteristics at Waves 1 and 2 separately. We reported percentages for categorical variables and means with standard deviations (SDs) for continuous variables.

We conducted fixed‐effects linear regression using the first‐difference approach (Allison 2009) to examine the association between affiliation with delinquent peers and cyberbullying perpetration. Fixed‐effects regression only uses the within‐subject variation and, therefore, can eliminate the confounding bias caused by all observed and unobserved time‐invariant variables. Specifically, the fixed‐effects regression can be illustrated as follows:

(1) $$Y_{it}=u_{t}+\beta X_{it}+\delta Z_{it}+\alpha_{i}+\varepsilon_{it}\;\;\;\;\;\;\;i=1,\text{…},n;\;t=1,\text{…},T,$$

where $Y_{it}$ is cyberbullying perpetration of individual *i* at time *t*, $X_{it}$ is affiliation with delinquent peers of individual *i* at time *t*, $u_{t}$ is the intercept, $Z_{it}$ is a vector of time‐variant control variables, $\alpha_{i\;}$ is the effects of observed and unobserved time‐invariant variables, and $\varepsilon_{it}$ is the random error term.

With two‐wave panel data (*T* = 2) and based on Equation (1), we obtained the following equations for *t* = 1 and *t* = 2, respectively,

$$Y_{i1}=u_{1}+\beta X_{i1}+\delta Z_{i1}+\alpha_{i}+\varepsilon_{i1}$$

$$Y_{i2}=u_{2}+\beta X_{i2}+\delta Z_{i2}+\alpha_{i}+\varepsilon_{i2}.$$

With the above two equations, we can obtain the following equation using the first‐difference method:

(2) $$Y_{i2}-Y_{i1}=\left(u_{2}-u_{1}\right)+\beta \left(X_{i2}-X_{i1}\right)+\delta \left(Z_{i2}-Z_{i1}\right)+\left(\varepsilon_{i2}-\varepsilon_{i1}\right),$$

where $\alpha_{i}$ in Equation (1) was ruled out, as it is time‐invariant. Hence, the fixed‐effects regression enabled us to eliminate the confounding bias caused by all observed and unobserved time‐invariant variables using within‐subject variations only. With Equation (2), we estimated β, the effect of affiliation with delinquent peers on cyberbullying perpetration after controlling for observed time‐variant variables and all observed and unobserved time‐invariant variables.

Furthermore, we specified an interaction term between affiliation with delinquent peers and student–teacher relationships in the fixed‐effects regression to examine the moderating effect of student–teacher relationships on the association between affiliation with delinquent peers and cyberbullying perpetration.

To examine the irreversible effect of affiliation with delinquent peers on cyberbullying perpetration, we conducted asymmetric fixed‐effects linear regression (Allison 2019). Similar to fixed‐effects regression, asymmetric fixed‐effects regression can control all the observed and unobserved time‐invariant confounders using the within‐subject variation. Furthermore, asymmetric fixed‐effects regression can decompose an independent variable into its increased and decreased components and, therefore, can examine whether both an increase and a decrease in the independent variable are associated with the dependent variable.

Using the two‐wave panel data in this study (*T* = 2), we can illustrate the asymmetric fixed‐effects regression as follows. First, ${\;X}_{it}$ is still affiliation with delinquent peers of individual *i* at time *t*. Then, we used $X_{i}^{+}$ and $X_{i}^{-}$ to indicate the increased and decreased affiliation with delinquent peers of individual *i* from *t* = 1 to *t* = 2, respectively, in which

$$X_{i}^{+}=X_{i2}-X_{i1}\;\text{if}\;X_{i2}-X_{i1}>0,\text{otherwise}\;0,$$

$$X_{i}^{-}=-\left(X_{i2}-X_{i1}\right)\;\text{if}\;X_{i2}-X_{i1}>0,\text{otherwise}\;0,$$

With $X_{i}^{+}$ and $X_{i}^{-}$ being defined, we specified the asymmetric fixed‐effects model using the first‐difference approach as follows:

(3) $$Y_{i2}-Y_{i1}=\left(u_{2}-u_{1}\right)+\beta^{+}X_{i}^{+}+\beta^{-}X_{i}^{-}+\delta \left(Z_{i2}-Z_{i1}\right)+\left(\varepsilon_{i2}-\varepsilon_{i1}\right).$$

Equation (3) is the same as Equation (2) except that ${\;X}_{it}$ in Equation (2) is decomposed into $X_{i}^{+}$ and $X_{i}^{-}$ in Equation (3). With Equation (3), we estimated $\beta^{+}$, the effect of increased affiliation with delinquent peers on cyberbullying perpetration, and $\beta^{-}$, the effect of decreased affiliation with delinquent peers on cyberbullying perpetration while controlling for observed time‐variant variables and all observed and unobserved time‐invariant variables.

Using the above approach, we also divided student–teacher relationships into its positive and negative components, namely, improved and worsened student–teacher relationships.

We included control variables in the regression analysis. Additionally, to handle the missing data issue, we performed multiple imputation by chained equations (White et al. 2011). We generated 20 imputed datasets and conducted regression analysis based on the imputed datasets. The Survey and Behavioral Research Committee of the Chinese University of Hong Kong approved this survey. No artificial intelligence tools or technologies were used in this study.

## 3 Results

### 3.1 Sample Characteristics

We reported the sample characteristics in Table 1. In terms of sex, 58.29% of the participants were female and 41.71% were male. Additionally, 32.87% of the participants were in Grade 7, 33.43% were in Grade 8%, and 33.71% were in Grade 9.

**TABLE 1:** Sample characteristics of junior high school students (Grades 7–9) in Hong Kong at Wave 1 and Wave 2.

	Wave 1 (*N* = 356)	Wave 2 (*N* = 356)
	Mean ± SD/Column %	Mean ± SD/Column %
Sex ^a^		
Female	58.29	58.29
Male	41.71	41.71
Grade ^b^		
Grade 7	32.87	32.87
Grade 8	33.43	33.43
Grade 9	33.71	33.71
Parental marital status ^c^		
Married	76.76	76.22
Divorced/separated/others	23.24	23.78
Family economic status ^d^		
Poor/average	71.38	69.82
Comfort/rich	28.62	30.18
Cyberbullying perpetration ^e^	6.35 ± 1.07	6.35 ± 1.74
Affiliation with delinquent peers ^f^	0.97 ± 1.64	1.06 ± 1.97
Student–teacher relationships ^g^	14.00 ± 2.26	14.08 ± 2.29

*Note:* Missing data (two waves in total): ^a^ 12; ^b^ 0; ^c^ 57; ^d^ 59^; e^ 55; ^f^ 55; ^g^ 15.

### 3.2 Associations Between Affiliation With Delinquent Peers, Student–Teacher Relationships, and Cyberbullying Perpetration

We reported the results based on fixed‐effects regression using Models 1a and 1b in Table 2. Model 1a shows that affiliation with delinquent peers was positively associated with cyberbullying perpetration (*β* = 0.17; 95% CI: 0.07, 0.27); that is, an increase in affiliation with delinquent peers was associated with increased cyberbullying perpetration; alternatively, a decrease in affiliation with delinquent peers was associated with decreased cyberbullying perpetration. The above interpretation was exactly based on the traditional *symmetric* effect assumption. Additionally, Model 1a shows that student–teacher relationships was not significantly associated with cyberbullying perpetration.

**TABLE 2:** The associations of affiliation with delinquent peers and student‐teacher relationships with cyberbullying perpetration (*N* = 356).

	FE		Asymmetric FE	
	Model 1a	Model 1b	Model 2a	Model 2b
	*β* (95% CI)	*β* (95% CI)	*β* (95% CI)	*β* (95% CI)
Affiliation with delinquent peers	0.17^**^ (0.07, 0.27)	0.17^**^ (0.07, 0.27)	—	—
Student‐teacher relationships	−0.02 (−0.13, 0.08)	−0.02 (−0.13, 0.09)	—	—
Affiliation with delinquent peers × student–teacher relationships	—	−0.01 (−0.06, 0.05)	—	—
Increased affiliation with delinquent peers (A)	—	—	0.22^**^ (0.08, 0.36)	0.44^**^ (0.18, 0.71)
Decreased affiliation with delinquent peers (B)	—	—	−0.09 (−0.27, 0.09)	−0.08 (−0.26, 0.09)
Improved student–teacher relationships (C)	—	—	−0.04 (−0.22, 0.14)	0.06 (−0.14, 0.26)
Worsened student–teacher relationships (D)	—	—	0.01 (−0.19, 0.21)	0.09 (−0.13, 0.31)
A × C	—	—	—	−0.13^*^ (−0.24, −0.02)
A × D	—	—	—	−0.14 (−0.34, 0.06)

*Note:* The fixed‐effects models and asymmetric fixed‐effects models were estimated by the first‐difference method. Time‐variant controls include parental marital status and subjective family economic status. Observed time‐invariant controls include sex and grade. The data were imputed using multiple imputation by chained equations.

Abbreviations: CI = confidence interval; FE = fixed‐effects models.

^*^ *p* < 0.05.

^**^ *p* < 0.01.

***p < 0.001.

Moreover, we added an interaction term between affiliation with delinquent peers and student–teacher relationships in the fixed‐effects regression shown using Model 1b. The interaction term was not statistically significant (Model 1b), indicating that we had no evidence to support the moderating effect of student–teacher relationships on the association between affiliation with delinquent peers and cyberbullying perpetration. Again, the above non‐significant moderating effect of student–teacher relationships was based on the *symmetric* effect assumption.

Furthermore, we reported the results based on asymmetric fixed‐effects regression using Models 2a and 2b in Table 2. Model 2a shows that increased affiliation with delinquent peers was associated with increased cyberbullying perpetration (*β* = 0.22; 95% CI: 0.08, 0.36), while decreased affiliation with delinquent peers was not significantly associated with cyberbullying perpetration. Additionally, neither improved nor worsened student–teacher relationships was significantly associated with cyberbullying perpetration.

To examine whether student–teacher relationships moderated the association between increased affiliation with delinquent peers and increased cyberbullying perpetration, we added the two interaction terms to Model 2b: (1) increased affiliation with delinquent peers interacted with improved student–teacher relationships (A × C) and (2) increased affiliation with delinquent peers interacted with worsened student–teacher relationships (A × D). Model 2b shows that the interaction term between increased affiliation with delinquent peers and improved student–teacher relationships (A × C) was significant and negative (*β* = −0.13; 95% CI: −0.24, −0.02), meaning that with a higher level of improvement in student–teacher relationships, the effect of increased affiliation with delinquent peers on cyberbullying perpetration became weaker. By contrast, the interaction term between increased affiliation with delinquent peers and worsened student–teacher relationships (A × D) was not statistically significant.

However, students who are already more rule‐abiding may naturally form stronger bonds with teachers, which may introduce selection bias. Specifically, in our study, the results could be biased due to the fact that students with improved student–teacher relationships from Wave 1 to Wave 2 were more likely to be those who had decreased cyberbullying perpetration and/or decreased affiliation with delinquent peers from Wave 1 to Wave 2. In other words, if changes in student–teacher relationships were correlated with changes in cyberbullying perpetration and/or those having decreased affiliation with delinquent peers, there could be selection bias. However, our results showed that the correlations of changes in student–teacher relationships with changes in cyberbullying perpetration (*r* = 0.01, *p* = 0.91) and changes in affiliation with delinquent peers (*r* = 0.03, *p* = 0.63) were extremely weak and not statistically significant. Therefore, the likelihood that we ran into the above‐mentioned selection bias was low.

## 4 Discussion

To our knowledge, this is the first study to examine the reversibility/irreversibility of the effect of affiliation with delinquent peers on cyberbullying perpetration. We found that based on the traditional *symmetric* effect assumption, an increase in affiliation with delinquent peers was associated with increased cyberbullying perpetration, which can essentially be interpreted as indicating that a reduction in affiliation with delinquent peers was associated with reduced cyberbullying perpetration. However, after we decomposed affiliation with delinquent peers into its increased and decreased components using *asymmetric* fixed‐effects regression, we found that increased affiliation with delinquent peers was associated with increased cyberbullying perpetration even after controlling for unobserved time‐invariant confounders, whereas decreased affiliation with delinquent peers was not associated with cyberbullying perpetration. Additionally, if, again, we assume the effect of student–teacher relationships was *symmetric*, we might find that student–teacher relationships did not moderate the association between affiliation with delinquent peers and cyberbullying perpetration. However, after further decomposing student–teacher relationships into its positive and negative components, we found that improving student–teacher relationships (the positive component) could buffer the adverse effect of increased affiliation with delinquent peers on cyberbullying perpetration.

Taken together, our results suggest that the traditional *symmetric* effect assumption in social science might lead to the erroneous conclusion that decreasing affiliation with delinquent peers could decrease cyberbullying perpetration. Additionally, the traditional *symmetric* effect assumption might hinder our understanding of moderating factors such as the student–teacher relationships in this study that can serve to protect individuals from the adverse effect of affiliation with delinquent peers on cyberbullying perpetration. By contrast, breaking the assumption of the *symmetric* effect could further advance our knowledge in the field of delinquent peer influence on delinquency.

### 4.1 Influence of Affiliation With Delinquent Peers on Cyberbullying Perpetration

Previous theories (Akers 1973; Akers et al. 1979; Mead 1934; Sutherland 1947) have posited that affiliation with delinquent peers can lead individuals to engage in delinquent behaviors, and studies have shown evidence supporting these theories in the offline setting (Akers et al. 1979; Chen, Yang, Lin, et al. 2023; Giletta et al. 2021; Grant et al. 2019; Haynie 2001; Monahan et al. 2009; Rodriguez et al. 2019). We examined the effect of affiliation with delinquent peers in the online setting and provided evidence to support the theories (Akers 1973; Akers et al. 1979; Mead 1934; Sutherland 1947) in the online setting. Additionally, compared with previous studies related to affiliation with delinquent peers and cyber delinquency (Guo 2021; Kim et al. 2022; X. Wang et al. 2025), we provided more solid evidence by eliminating the effect of unobserved time‐invariant confounders.

Furthermore, we found that although increased affiliation with delinquent peers was a risk factor for cyberbullying perpetration, reducing the risk factor might not reduce cyberbullying perpetration. In other words, the effect of increased affiliation with delinquent peers on increased cyberbullying perpetration might not be reversible by reducing affiliation with delinquent peers. The results suggest that affiliation with delinquent peers has a long‐term effect on cyberbullying perpetration (M. Wang et al. 2022). Additionally, as differential association theory (Sutherland 1947) posits, affiliation with delinquent peers causes delinquency by fostering favorable attitudes toward delinquency (McGloin and Thomas 2019). Hence, even if affiliation with delinquent peers is decreased, the favorable attitudes toward delinquency caused by affiliation with delinquent peers might not be reduced. Therefore, decreased affiliation with delinquent peers was not associated with reduced cyberbullying perpetration.

Additionally, according to social learning theory (Akers 1973; Akers et al. 1979), the process of learning delinquency from delinquent peers involves operant (instrumental) conditioning; that is, the learned behaviors are also influenced by positive or negative stimuli, such as rewards or punishments. In the case of cyberbullying perpetration, the previous cyberbullying perpetration experience might be a positive stimulus for further cyberbullying perpetration as it was shown that previous cyberbullying perpetration was associated with future cyberbullying perpetration (M. Wang et al. 2022), and decreasing affiliation with delinquent peers could not remove the stimuli of previous cyberbullying perpetration; therefore, even if affiliation with delinquent peers decreased, cyberbullying perpetration did not. Furthermore, decreased affiliation with delinquent peers per se is not as negative a stimulus as a punishment; therefore, it could not modify cyberbullying perpetration.

Moreover, drawing on symbolic interactionism (Goffman 1959; Mead 1934), interactions with delinquent peers may lead to the expectation that delinquency is favorable. By contrast, decreased interactions with delinquent peers might not change the favorable attitude toward delinquency; hence, individuals may continue to be delinquent in the absence of peer pressure (Mulligan 1960). Additionally, decreased interactions with delinquent peers may not necessarily mean more interactions with non‐delinquent peers, which can encourage unfavorable attitudes toward delinquency. Hence, individuals may not be guided by unfavorable attitudes toward delinquency even if their affiliation with delinquent peers decreases and, thus, their delinquent behaviors are not modified. In this sense, decreased affiliation with delinquent peers does not decrease cyberbullying perpetration.

### 4.2 Moderating Effect of Improved Student–Teacher Relationships

We found that although reducing affiliation with delinquent peers may not reduce cyberbullying perpetration caused by increased affiliation with delinquent peers, improving student–teacher relationships could mitigate the effect of increased affiliation with delinquent peers on increased cyberbullying perpetration. Therefore, teachers play a vital role in the prevention of cyberbullying perpetration for students at risk of delinquent peer influence.

According to differential association theory (Sutherland 1947), affiliation with delinquent peers leads to favorable attitudes toward delinquency, which in turn results in delinquent behaviors (McGloin and Thomas 2019). In this sense, the above moderating effect of improved student–teacher relationships suggests that these relationships counteracted students' favorable attitudes toward delinquency caused by delinquent peers, thereby mitigating the effect of increased affiliation with delinquent peers on increased cyberbullying perpetration. Additionally, improved student–teacher relationships may be accompanied by more student–teacher interaction. Drawing on symbolic interactionism (Goffman 1959; Mead 1934), student–teacher interactions could affect students' expectations of others' reactions to their behaviors. In the scenario of delinquency, more interactions with teachers may enhance the expectation that delinquency is met with disapproval in society, which may reduce the effect of affiliation with delinquent peers on delinquency.

Furthermore, improved student–teacher relationships may indicate more teacher support for students, which could enhance students' self‐control (Zhang et al. 2021). Self‐control, subsequently, can buffer peer influence on delinquency (Meldrum et al. 2013). Therefore, student–teacher relationships could mitigate the effect of increased affiliation with delinquent peers on increased cyberbullying perpetration.

### 4.3 Implications

The results of this study imply that increased affiliation with delinquent peers is a risk factor for cyberbullying perpetration; however, the risk might not be reduced or removed by reducing students' affiliation with delinquent peers. Therefore, interventions to prevent or reduce cyberbullying perpetration by reducing affiliation with delinquent peers might not be as effective as expected.

However, improving student–teacher relationships could be the means of interventions and policies to reduce cyberbullying perpetration since it can mitigate the effect of increased affiliation with delinquent peers on increased cyberbullying perpetration. Therefore, to prevent and reduce cyberbullying perpetration, schools could consider introducing programs that can improve student–teacher relationships. For instance, schools can provide brief training for teachers using the Establish‐Maintain‐Restore (EMR) approach, which was shown to be effective in improving student–teacher relationships in the United States (Duong et al. 2019). The EMR approach involves 3‐h training and ongoing implementation supports and can help teachers enhance their ability to foster relationships with students. Additionally, to improve teachers' relationships with students who engage in cyberbullying perpetration, schools could consider interventions such as Key2Teach, which is a teacher‐focused coaching intervention that can help teachers improve their relationships with students who exhibit externalizing problem behaviors (Hoogendijk et al. 2020).

Furthermore, at the policy level, education authorities may consider providing budgets specifically for interventions in student–teacher relationships. Additionally, education authorities may contemplate offering regular training for teachers to enhance their skills in fostering relationships with students, in addition to enhancing their teaching skills.

### 4.4 Limitations

Although we employed fixed‐effects models to control unobserved time‐invariant confounders, we could not eliminate the effects of unobserved time‐variant confounders. If unobserved time‐variant confounders affect both affiliation with delinquent peers and cyberbullying perpetration, the results could be biased. We only included parental marital status and subjective family economic status as time‐variant variables, leaving other time‐variant variables unobserved and uncontrolled. Thus, future studies may consider controlling more time‐variant confounders. Additionally, we could not rule out the possibility of reverse causality; that is, students who engaged in cyberbullying perpetration may be more likely to make friends with delinquent students. Previous research established a temporal association between affiliation with delinquent peers and cyberbullying perpetration (X. Wang et al. 2025). However, the approach of establishing a temporal association is subject to confounding bias caused by unobserved factors. Therefore, we call for future studies to employ causal inference identification strategies, such as instrumental variable estimation, to provide more solid evidence for the causal role of affiliation with delinquent peers in cyberbullying perpetration. Furthermore, the sample in this study was from Hong Kong, which may limit the generalizability of the results. Therefore, future studies using various samples are needed to examine the irreversible effect of increased affiliation with delinquent peers on cyberbullying perpetration. Last, because we only used two waves of follow‐up data and the time interval was 9 months, we might have been unable to observe enough within‐subject variation of affiliation with delinquent peers. Future studies may employ more waves of follow‐up data with longer time intervals to examine the association between decreased affiliation with delinquent peers and cyberbullying perpetration.

## 5 Conclusion

Affiliation with delinquent peers is a risk factor for cyberbullying perpetration. Furthermore, the effect of increased affiliation with delinquent peers on increased cyberbullying perpetration may be irreversible in that reducing affiliation with delinquent peers could not reduce cyberbullying perpetration. Improving student–teacher relationships could mitigate the effect of increased affiliation with delinquent peers on increased cyberbullying perpetration. Therefore, policies and interventions should aim to improve student–teacher relationships to buffer the adverse effects of increased affiliation with delinquent peers on cyberbullying perpetration.

## Author Contributions

Y.H. was responsible for the literature review, study design, data analysis, data interpretation, and drafting the manuscript. J‐K.C. oversaw the whole study and was responsible for the literature review, study design, data collection, data interpretation, write‐up of the manuscript, and funding acquisition.

## Ethics Statement

The survey, informed consent, research procedures, and ethical issues were reviewed and approved by the Survey and Behavioral Research Committee at the Chinese University of Hong Kong.

## Conflicts of Interest

The authors declare no conflicts of interest.

## Data Availability

The data that support the findings of this study are available from the corresponding author upon reasonable request.
